# Dichlorido{*N*′-[(pyridin-2-yl)methyl­idene-κ*N*]acetohydrazide-κ^2^ *N*′,*O*}copper(II)

**DOI:** 10.1107/S1600536811049671

**Published:** 2011-11-30

**Authors:** Amitabha Datta, Shiann-Cherng Sheu, Pei-Hsin Liu, Jui-Hsien Huang

**Affiliations:** a Department of Chemistry, National Changhua University of Education, Changhua 50058, Taiwan; b Department of Occupational Health and Safety, Chang Jung Christian University, Tainan City 71101, Taiwan

## Abstract

In the title compound, [CuCl_2_(C_8_H_9_N_3_O)], the Cu^II^ atom has a distorted square-pyramidal CuCl_2_N_2_O coordination geometry. The tridentate acetohydrazide ligand occupies three basal positions, the fourth basal position being defined by a chloride anion at a distance of 2.2116 (6) Å. The second chloride anion is in the apical position and forms a longer Cu—Cl distance of 2.4655 (7) Å. Inter­molecular N—H⋯Cl hydrogen bonds are present in the crystal, leading to the formation of chains along [10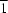].

## Related literature

For related copper(II) complexes with a similar tridentate ligand, see: Sen *et al.* (2005 ▶, 2007**a* ▶,b* ▶), Ray *et al.* (2008**a* ▶,b* ▶), Recio Despaigne *et al.* (2009 ▶); Datta *et al.* (2010**a* ▶,b* ▶, 2011 ▶).
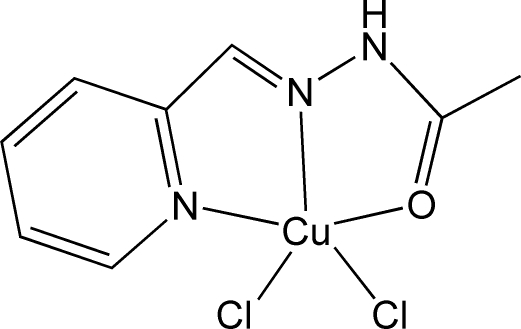


## Experimental

### 

#### Crystal data

[CuCl_2_(C_8_H_9_N_3_O)]
*M* *_r_* = 297.62
Monoclinic, 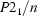

*a* = 6.8326 (12) Å
*b* = 15.137 (3) Å
*c* = 10.689 (3) Å
β = 95.664 (13)°
*V* = 1100.0 (4) Å^3^
*Z* = 4
Mo *K*α radiation
μ = 2.45 mm^−1^
*T* = 150 K
0.40 × 0.25 × 0.25 mm

#### Data collection

Bruker APEXII CCD diffractometer
Absorption correction: multi-scan (*SADABS*; Sheldrick, 1996 ▶) *T*_min_ = 0.485, *T*_max_ = 0.543
9791 measured reflections
2836 independent reflections
2378 reflections with *I* > 2σ(*I*)
*R*_int_ = 0.024

#### Refinement

*R*[*F* ^2^ > 2σ(*F* ^2^)] = 0.024
*wR*(*F* ^2^) = 0.070
*S* = 1.04
2836 reflections
137 parameters
H-atom parameters constrained
Δρ_max_ = 0.31 e Å^−3^
Δρ_min_ = −0.35 e Å^−3^

### 

Data collection: *APEX2* (Bruker, 2007 ▶); cell refinement: *SAINT* (Bruker, 2007 ▶); data reduction: *SAINT*; program(s) used to solve structure: *SHELXS97* (Sheldrick, 2008 ▶); program(s) used to refine structure: *SHELXL97* (Sheldrick, 2008 ▶); molecular graphics: *XP* in *SHELXTL* (Sheldrick, 2008 ▶); software used to prepare material for publication: *SHELXTL*.

## Supplementary Material

Crystal structure: contains datablock(s) I, global. DOI: 10.1107/S1600536811049671/wm2553sup1.cif

Structure factors: contains datablock(s) I. DOI: 10.1107/S1600536811049671/wm2553Isup2.hkl

Additional supplementary materials:  crystallographic information; 3D view; checkCIF report

## Figures and Tables

**Table 1:** Hydrogen-bond geometry (Å, °)

*D*—H⋯*A*	*D*—H	H⋯*A*	*D*⋯*A*	*D*—H⋯*A*
N3—H3*A*⋯Cl3^i^	0.88	2.21	3.0799 (16)	170

Symmetry code: (i) 
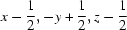
.

**Fig. 1.**
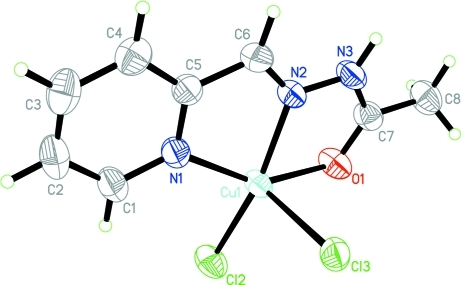
The molecular structure of the title complex, showing 50% displacement ellipsoids.

**Fig. 2.**
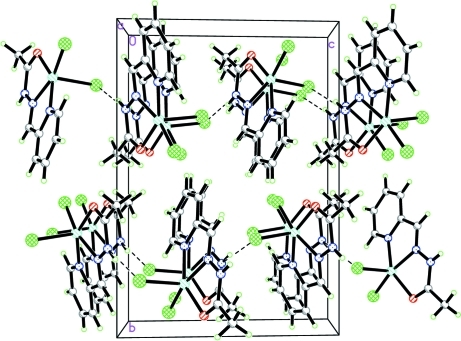
Packing diagram of the title compound as viewed down the a axis. Intermolecular N—H···Cl hydrogen bonds are shown as dashed lines.

## supplementary crystallographic information

### Comment

In the title compound (Fig. 1), the copper(II) ion exhibits a distorted square pyramidal geometry. The *N*'-(pyridine-2-ylmethylene)acetohydrazide ligand is in its keto form as indicated by the short C—O distance of 1.235 (2) Å and defines three of the basal positions *via* the pyridyl N, imine N, and keto O atoms. The fourth basal position is provided by a chloride anion, *trans* to the imine N atom. Another chloride ligand occupies the apical position. The two Cu—Cl distances are unequal in length. The chloride ligand in the apical position forms a long Cu—Cl bond of 2.4655 (7) Å, whereas the Cu—Cl bond to the basal chloride anion is much shorter (2.2116 (6) Å).

Classical intermolecular hydrogen bonds of the type N—H···Cl are present along the [101] direction (Fig. 2), leading to the formation of chains.

The structure of a copper(II) dichloride complex with a similar tridentate hydrazone ligand has been reported in the literature (Datta, *et al.*, 2011). For other related copper(II) complexes with similar tridentate ligands, see: Sen *et al.* (2005, 2007**a*,b*), Ray *et al.* (2008**a*,b*), Recio Despaigne *et al.* (2009); Datta *et al.* (2010**a*,b*).

### Experimental

The tridentate acetohydrazide ligand precursor was prepared according to the literature procedure (Ray *et al.*, 2008*b*). To a hot methanolic solution (20 ml) of anhydrous CuCl_2_ (0.134 g, 1.0 mmol), the ligand (0.163 g, 1.0 mmol) was added, which produced immediately an intensely green solution. The mixture was then heated to boiling. On cooling to room temperature and after slow evaporation of the green solution, dark green rectangular shaped single crystals of the complex were separated out after 3 days. The crystals were filtered off and washed with water and dried in air.

### Refinement

Carbon- and nitrogen-bound H-atoms were placed in calculated positions (C—H 0.95 to 0.98 Å and N—H 0.88 Å) and were included in the refinement in the riding model approximation, with *U*_iso_(H) set to 1.2 and 1.5 times *U*_eq_(C,*N*).

### Crystal data

**Table:**

[CuCl_2_(C_8_H_9_N_3_O)]	*F*(000) = 596
*M**_r_* = 297.62	*D*_x_ = 1.797 Mg m^−^^3^
Monoclinic, *P*2_1_/*n*	Mo *K*α radiation, λ = 0.71073 Å
Hall symbol: -P 2yn	Cell parameters from 4370 reflections
*a* = 6.8326 (12) Å	θ = 3.3–28.6°
*b* = 15.137 (3) Å	µ = 2.45 mm^−^^1^
*c* = 10.689 (3) Å	*T* = 150 K
β = 95.664 (13)°	Rectangular, green
*V* = 1100.0 (4) Å^3^	0.40 × 0.25 × 0.25 mm
*Z* = 4	

### Data collection

**Table:**

Bruker APEXII CCD diffractometer	2836 independent reflections
Radiation source: fine-focus sealed tube	2378 reflections with *I* > 2σ(*I*)
graphite	*R*_int_ = 0.024
phi and ω scans	θ_max_ = 28.8°, θ_min_ = 2.3°
Absorption correction: multi-scan (*SADABS*; Sheldrick, 1996)	*h* = −9→9
*T*_min_ = 0.485, *T*_max_ = 0.543	*k* = −19→20
9791 measured reflections	*l* = −14→6

### Refinement

**Table:**

Refinement on *F*^2^	Primary atom site location: structure-invariant direct methods
Least-squares matrix: full	Secondary atom site location: difference Fourier map
*R*[*F*^2^ > 2σ(*F*^2^)] = 0.024	Hydrogen site location: inferred from neighbouring sites
*wR*(*F*^2^) = 0.070	H-atom parameters constrained
*S* = 1.04	*w* = 1/[σ^2^(*F*_o_^2^) + (0.0373*P*)^2^ + 0.2632*P*] where *P* = (*F*_o_^2^ + 2*F*_c_^2^)/3
2836 reflections	(Δ/σ)_max_ = 0.001
137 parameters	Δρ_max_ = 0.31 e Å^−^^3^
0 restraints	Δρ_min_ = −0.35 e Å^−^^3^

### Special details

**Table:**

Geometry. All e.s.d.'s (except the e.s.d. in the dihedral angle between two l.s. planes) are estimated using the full covariance matrix. The cell e.s.d.'s are taken into account individually in the estimation of e.s.d.'s in distances, angles and torsion angles; correlations between e.s.d.'s in cell parameters are only used when they are defined by crystal symmetry. An approximate (isotropic) treatment of cell e.s.d.'s is used for estimating e.s.d.'s involving l.s. planes.
Refinement. Refinement of *F*^2^ against ALL reflections. The weighted *R*-factor *wR* and goodness of fit *S* are based on *F*^2^, conventional *R*-factors *R* are based on *F*, with *F* set to zero for negative *F*^2^. The threshold expression of *F*^2^ > σ(*F*^2^) is used only for calculating *R*-factors(gt) *etc*. and is not relevant to the choice of reflections for refinement. *R*-factors based on *F*^2^ are statistically about twice as large as those based on *F*, and *R*- factors based on ALL data will be even larger.

### Fractional atomic coordinates and isotropic or equivalent isotropic displacement parameters (Å^2^)

**Table:**

	*x*	*y*	*z*	*U*_iso_*/*U*_eq_	
Cu1	0.24493 (3)	0.323357 (14)	0.19561 (2)	0.03400 (8)	
Cl2	0.47106 (7)	0.41980 (4)	0.26879 (5)	0.04816 (13)	
Cl3	0.05430 (7)	0.30589 (3)	0.37823 (4)	0.04052 (12)	
N1	0.4124 (2)	0.21177 (10)	0.19800 (14)	0.0346 (3)	
C5	0.3171 (3)	0.14208 (12)	0.13949 (16)	0.0345 (4)	
C4	0.4016 (3)	0.05932 (13)	0.13604 (19)	0.0443 (4)	
H4	0.3316	0.0115	0.0952	0.053*	
C1	0.5949 (3)	0.19982 (15)	0.25159 (19)	0.0427 (4)	
H1	0.6633	0.2483	0.2918	0.051*	
C2	0.6879 (3)	0.11807 (16)	0.2502 (2)	0.0517 (5)	
H2	0.8185	0.1112	0.2885	0.062*	
C3	0.5901 (3)	0.04753 (15)	0.1932 (2)	0.0524 (5)	
H3	0.6511	−0.0088	0.1931	0.063*	
C6	0.1226 (3)	0.16357 (13)	0.07736 (18)	0.0385 (4)	
H6	0.0400	0.1212	0.0330	0.046*	
N2	0.0738 (2)	0.24430 (10)	0.08798 (14)	0.0340 (3)	
N3	−0.0947 (2)	0.28140 (11)	0.03418 (14)	0.0391 (3)	
H3A	−0.1916	0.2506	−0.0048	0.047*	
O1	0.0387 (2)	0.40997 (9)	0.10589 (13)	0.0437 (3)	
C7	−0.0995 (3)	0.37105 (13)	0.04643 (16)	0.0377 (4)	
C8	−0.2756 (3)	0.41705 (15)	−0.0143 (2)	0.0498 (5)	
H8A	−0.3316	0.4550	0.0474	0.075*	
H8B	−0.3737	0.3733	−0.0465	0.075*	
H8C	−0.2380	0.4533	−0.0841	0.075*	

### Atomic displacement parameters (Å^2^)

**Table:**

	*U*^11^	*U*^22^	*U*^33^	*U*^12^	*U*^13^	*U*^23^
Cu1	0.02943 (13)	0.03512 (13)	0.03591 (13)	−0.00313 (9)	−0.00455 (8)	−0.00127 (8)
Cl2	0.0370 (2)	0.0471 (3)	0.0583 (3)	−0.0125 (2)	−0.0054 (2)	−0.0031 (2)
Cl3	0.0328 (2)	0.0516 (3)	0.0369 (2)	0.0043 (2)	0.00207 (16)	0.00244 (18)
N1	0.0301 (7)	0.0391 (8)	0.0342 (7)	−0.0002 (7)	0.0019 (6)	0.0013 (6)
C5	0.0336 (9)	0.0372 (9)	0.0327 (8)	−0.0003 (8)	0.0042 (7)	0.0003 (7)
C4	0.0477 (11)	0.0359 (10)	0.0504 (11)	0.0001 (9)	0.0104 (9)	0.0017 (8)
C1	0.0306 (9)	0.0526 (11)	0.0441 (10)	−0.0015 (9)	−0.0010 (7)	0.0026 (9)
C2	0.0347 (10)	0.0626 (14)	0.0573 (12)	0.0107 (10)	0.0017 (9)	0.0118 (11)
C3	0.0501 (12)	0.0462 (12)	0.0621 (13)	0.0136 (10)	0.0117 (10)	0.0111 (10)
C6	0.0375 (10)	0.0394 (10)	0.0380 (9)	−0.0045 (8)	0.0003 (7)	−0.0056 (7)
N2	0.0298 (7)	0.0398 (8)	0.0312 (7)	−0.0005 (6)	−0.0035 (5)	−0.0016 (6)
N3	0.0320 (8)	0.0430 (9)	0.0396 (8)	−0.0006 (7)	−0.0092 (6)	−0.0028 (6)
O1	0.0435 (7)	0.0380 (7)	0.0466 (7)	−0.0043 (6)	−0.0103 (6)	0.0044 (6)
C7	0.0365 (9)	0.0445 (10)	0.0313 (8)	0.0000 (8)	−0.0013 (7)	0.0054 (7)
C8	0.0432 (11)	0.0520 (12)	0.0516 (12)	0.0059 (10)	−0.0086 (9)	0.0072 (9)

### Geometric parameters (Å, °)

**Table:**

Cu1—N2	1.9638 (15)	C2—C3	1.370 (3)
Cu1—N1	2.0390 (16)	C2—H2	0.9500
Cu1—O1	2.0872 (14)	C3—H3	0.9500
Cu1—Cl2	2.2116 (6)	C6—N2	1.275 (2)
Cu1—Cl3	2.4655 (7)	C6—H6	0.9500
N1—C1	1.332 (2)	N2—N3	1.357 (2)
N1—C5	1.359 (2)	N3—C7	1.364 (3)
C5—C4	1.381 (3)	N3—H3A	0.8800
C5—C6	1.462 (3)	O1—C7	1.235 (2)
C4—C3	1.381 (3)	C7—C8	1.484 (3)
C4—H4	0.9500	C8—H8A	0.9800
C1—C2	1.392 (3)	C8—H8B	0.9800
C1—H1	0.9500	C8—H8C	0.9800
			
N2—Cu1—N1	78.67 (6)	C1—C2—H2	120.2
N2—Cu1—O1	77.16 (6)	C2—C3—C4	119.2 (2)
N1—Cu1—O1	151.48 (6)	C2—C3—H3	120.4
N2—Cu1—Cl2	164.60 (5)	C4—C3—H3	120.4
N1—Cu1—Cl2	99.83 (5)	N2—C6—C5	114.00 (16)
O1—Cu1—Cl2	99.45 (4)	N2—C6—H6	123.0
N2—Cu1—Cl3	93.90 (5)	C5—C6—H6	123.0
N1—Cu1—Cl3	103.94 (4)	C6—N2—N3	125.27 (16)
O1—Cu1—Cl3	92.62 (5)	C6—N2—Cu1	119.37 (13)
Cl2—Cu1—Cl3	101.30 (2)	N3—N2—Cu1	115.30 (12)
C1—N1—C5	118.57 (17)	N2—N3—C7	113.47 (15)
C1—N1—Cu1	128.19 (14)	N2—N3—H3A	123.3
C5—N1—Cu1	113.21 (12)	C7—N3—H3A	123.3
N1—C5—C4	122.24 (18)	C7—O1—Cu1	112.58 (12)
N1—C5—C6	114.14 (16)	O1—C7—N3	119.99 (17)
C4—C5—C6	123.58 (18)	O1—C7—C8	123.20 (19)
C3—C4—C5	118.6 (2)	N3—C7—C8	116.80 (17)
C3—C4—H4	120.7	C7—C8—H8A	109.5
C5—C4—H4	120.7	C7—C8—H8B	109.5
N1—C1—C2	121.7 (2)	H8A—C8—H8B	109.5
N1—C1—H1	119.2	C7—C8—H8C	109.5
C2—C1—H1	119.2	H8A—C8—H8C	109.5
C3—C2—C1	119.7 (2)	H8B—C8—H8C	109.5
C3—C2—H2	120.2		

### Hydrogen-bond geometry (Å, °)

**Table:**

*D*—H···*A*	*D*—H	H···*A*	*D*···*A*	*D*—H···*A*
N3—H3A···Cl3^i^	0.88	2.21	3.0799 (16)	170.

Symmetry codes: (i) *x*−1/2, −*y*+1/2, *z*−1/2.
